# Polysomnography in Transition: Reassessing Its Role in the Future of Sleep Medicine

**DOI:** 10.1111/jsr.70217

**Published:** 2025-10-10

**Authors:** Damien Leger, Carlotta Mutti, Alexandre Rouen, Liborio Parrino

**Affiliations:** ^1^ Université Paris Cité, VIFASOM, (UMR Vigilance Fatigue Sommeil et Santé Publique) Paris France; ^2^ APHP, Hôtel‐Dieu, Centre du Sommeil et de la Vigilance Paris France; ^3^ Sleep Disorders Center, Department of Neurosciences Parma University Hospital Parma Italy; ^4^ Mario Giovanni Terzano Interdepartmental Center for Sleep Disorders University of Parma Parma Italy

**Keywords:** automatic sleep staging, first night effect, inter scorer's reliability burden of sleep, polysomnography, wearables

## Abstract

PSG, a cornerstone diagnostic instrument in sleep medicine, is recommended for the diagnosis of numerous sleep disorders and might be used as a benchmark for evaluating therapeutical effectiveness. However, PSG has its limitations, and its usefulness in the future warrants reappraisal. First, it is a complex test that requires highly‐trained personnel to correctly place the electrodes, monitor the patient, and manually analyse the data. This constitutes a significant economic burden for both society and healthcare systems. PSG also presents few technical limitations: variability of data between nights and variable reliability of scoring between readers. The results given to patients are also limited to the macrostructure of sleep, risking the loss of important information that escapes detection when relying solely on polysomnographic evaluation based on macro sleep stages. The current sleep scoring guidelines raise some doubts about their ability to capture the dynamic and complex nature of human sleep in both clinical and physiological contexts. On the other hand, advanced PSG analysis can provide key information for diagnosis particularly through the microstructural analysis of NREM oscillatory pattern, characterisation of spindles and slow waves, eye movement density, spectrum analysis with hypnodensity and sleep propensity with odd ratio products (ORP). These elements provide a better understanding of the differences between insomnia and poor sleep perception. Furthermore, these methods take into account the dynamics of sleep states, going beyond the mere distinction of sleep into macro stages, which poorly reflects the dynamic nature of sleep itself and including in the assessment of sleep function all the complex associations that sleep itself has with autonomic and cardiorespiratory variables. These insights will transform the role of technicians and clinicians in PSG analysis, with a shift towards training in digital data analysis and algorithms to better inform patients about their PSG results.

## Graphical abstract

This figure summarizes the disadvantages (“Cons”) and advantages (“Pros”) of developing polysomnography in the future. Abbreviations: CAPS: cyclic alternative patterns; ORP: odd ratio products; PSG: polysomnography; REM: rapid eye movement sleep.

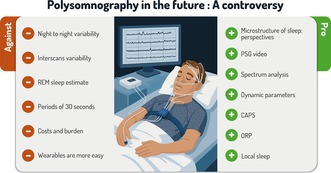

## 1 Introduction

It would be pointless to discuss sleep medicine today without mentioning polysomnography (PSG). PSG is the foundation of our knowledge and definition of sleep. The pioneers who identified rapid eye movement (REM) sleep, Aserinski, Dement, Kleitman and Jouvet, initially described the polysomnographic recordings of their relatives, who were fitted with electrodes in rudimentary research laboratories (Aserinsky and Kleitman 1979; Dement and Kleitman 1957; Jouvet 1965). Observing rapid brain activity despite muscle atonia, Jouvet referred to this as “paradoxical” sleep (Jouvet 1965). By identifying REMs occurring in bursts during the same stage, Aserinski and Dement defined REM sleep (Aserinsky and Kleitman 1979; Dement and Kleitman 1957). Then, little by little over the following decades, the Retschaffen and Kales sleep coding manual, published by the University of California, described the stages of sleep in detail in the manual (Rechtschaffen and Kales 1968). Curiously, despite constituting the majority of total sleep duration, non‐rapid eye movement (non‐REM) sleep continues to be defined in negative terms, implicitly positioning REM sleep as the primary or defining stage in the conceptual framework of sleep architecture. The first international sleep researchers pored over the pages of this large, flexible paper manual to refer to and define the sleep stages of their patients.

At that time, not so long ago, patients recorded by massive electroencephalographic (EEG) recording equipment slept under the continuous supervision of sleep technicians or doctors while hundreds of sheets of EEG paper were rolled out, resulting in a 10‐kg trace that had to be analysed page by hand by the doctor. Then the AASM manual for scoring sleep and associated rules published consensual and standardised recommendations (Iber et al. 2007; Berry et al. 2012, 2017).

It was also based on PSG that the main sleep disorders were initially defined and classified by successive international classifications of sleep disorders, including the later one ICSD‐3rd rev (International Classification of Sleep Disorders 2024). PSG is still a mandatory criteria for the definition of numerous sleep disorders (Boulos et al. 2019).

Since 2000, sleep medicine has therefore developed worldwide around PSG. Technological and digital gaps have been bridged, making this test much easier to record thanks to the miniaturisation of electronic components and sensors. Computerization allows for easier and largely assisted re‐reading of polysomnographic tracings.

Reimbursement for sleep disorders by social security systems typically requires objective diagnostic evidence, as those provided by PSG or polygraphic recording (e.g., obstructive sleep apnea (OSA) or periodic limb movement disorder). Efficacy of sleep‐promoting drugs need to be tested objectively with PSG according to the FDA and EMA guidelines (Food and Drug Administration: US Department of Health E and W 1997; European Medicines Agency 2011).

However, PSG remains a highly technical and difficult test to perform, time‐consuming compared to other sleep examinations (e.g., cardio‐respiratory monitoring), strongly prone to recording artefact, due to the location of sensors close to facial muscles, eyes, and cardio‐respiratory system. It requires specialised personnel, expensive equipment, and nights of observation, routinely performed in sleep laboratories. Recently, most tests have been performed on an outpatient basis, reducing the cost of the test, but this exposes the examination to possible quality degradation in the absence of technical staff to address sensor misplacement, and may render the recording hard to interpret in the case of nocturnal hyper‐motor events.

Looking to the future, it is therefore not unreasonable to ask the question: PSG: where are we headed in sleep medicine? Many believe that this deserves to be discussed.

PSG has indeed technical limitations and its analysis is subject to discrepancies between scorers. It is not always necessary for the diagnosis of all sleep disorders. It is expensive and can limit access to care.

However, it can also potentially provide much more information than is usually analysed manually. Through microstructural analysis or AI, it can provide information on sleep dynamics and allow for a better understanding of certain unresolved sleep disorders.

It is this controversy surrounding PSG that we wish to discuss here with a view to the future.

## 2 Technical Limitations in Current PSG Scoring

The rules for acquiring and interpreting PSG are the subject of an internationally recognised consensus published in the American Academy of Sleep Medicine's manual for scoring sleep and related events (AASM) (Iber et al. 2007; Berry et al. 2012, 2017). These rules enable sleep experts to be trained according to the same criteria worldwide. They also allow for the same care of patients with sleep disorders according to the same criteria recognised by specialists and health authorities. They have enabled consistency and growth in sleep medicine over the last few decades.

However, there are technical limitations associated with v‐PSG that are more or unanimously recognised by specialists.

### 2.1 Night‐To‐Night Variability in Sleep Patterns: Is One‐Night of PSG Recording Enough?

Patients themselves often question the reliability of a single night of polysomnography when they received the diagnosis of insomnia, sleep apnea or hypersomnia. This variability between two nights of PSG is well known in chronic insomnia disorder and it is described under the term “first night effect (FNE)”. During the first night of recording, sleep generally includes a greater sleep onset latency (SOL), more nocturnal awakenings, a decreased TST, more light sleep stages and decreased percentage of REM sleep and increased REM latency (Hu et al. 2024). This FNE is well recognised in pharmaceutical research on insomnia, to such an extent that both the US FDA and the European EMEA recommend two consecutive nights of PSG before and after each time point in a therapeutic trial to evaluate a new drug (Food and Drug Administration: US Department of Health E and W 1997; European Medicines Agency 2011). Typically, only the second night is taken into account. Interestingly, approximatively 40% of chronic insomnia patients presenting short sleep duration (< 6 h) at the first night therefore increased their TST on the following night (Cox et al. 2024).

Notably, even good sleepers tested undergo a FNE, limiting the informatively of a single night of recording in basically all sleep studies.

According to these premises, do we need to perform several nights of v‐PSG to reliably confirm a sleep diagnosis?

To answer this controversial question, it is worth noting an interesting Japanese study, comparing objective measurements, subjective complaints, and clinicians' opinions with respect to chronic insomnia, sleep deprivation, and OSA (Masaki et al. 2025). In this survey, 421 participants aged 20–79 years and their 1465 nights of recording were selected. Subjects included answered a general health questionnaire and underwent from 1 to 6 nights of PSG recording, inclusive of saturimetric profile. The authors, comparing the at‐home PSG with the in‐lab recording data, found some differences in sleep organisation, such as a longer total sleep time (TST) and higher sleep efficiency in the domestic setting that reasonably reduce the FNE and reflect the everyday life characteristics of sleep in a more reliable fashion. They also found that participants with moderate‐to‐severe sleep insufficiency overestimated their sleep duration, whereas those with subjective insomnia but with no instrumental confirmation underestimated it. Accordingly, they propose to subdivide insomnia patients into two groups: those with combined objective and subjective insomnia and those with only subjective insomnia. In this perspective sleep recording might be useful to phenotype insomnia patients, a topic that remains essential for improving diagnostic precision and enhancing our understanding of the different pathophysiological mechanisms underlying this condition. Some differences between home‐based and in‐laboratory PSG recordings are evident in their modulation of the FNE. The home environment, along with the absence of direct interaction with medical personnel during the recording night, likely reduces the psychological impact of the procedure on the patient. As a result, the first‐night recording in a home setting may be partially more reliable. Although with at‐home PSG recording the FNE are minimal, the existing between night variance cannot be totally ignored, especially among chronic insomnia patients. The coefficient of variation was particularly high for those judged to have objective insomnia, especially for TST, SE, and WASO. This means that there is a greater possibility of overestimate objective insomnia when only considering the first night. In fact, among those judged to have objective insomnia, 54.0% exhibited objective insomnia for the first time on the first night, 37.2% on the second night, 6.2% on the third night, and 2.6% on the fourth night, respectively. Conversely, among those with high apnea‐hypopnea index of 15–24, only 42.9% were found to have objective insomnia based on the first night, while this figure rose to 62.5% with multiple nights. Hence, depending on patients diagnosis, there is a high risk for either over or under‐estimation of sleep duration.

Another recent study looked at the reliability and repeatability of PSG combined with multi‐sleep latency test (MSLT) for the diagnosis of type 1 narcolepsy (NT1) and non‐narcoleptic hypersomnolence disorders in Denmark (Torstensen et al. 2023). According to this study, PSG data have good reliability and repeatability in subjects with NT1 with low hypocretin levels (acuity 0.88, reliability 0.80), but the same data are weak in the identification of patients with hypersomnolence disorders and normal hypocretin levels (reliability 0.3 and low accuracy).

Finally, a recent study (Strassberger et al. 2023) verified the stability of PSG parameters in different nights in patients affected by OSA. According to their results, OSA respiratory features (including mean AHI and ODI) and main endotypes are relatively stable in consecutive nights, while night characteristics change: specifically, patients slept longer on the second night, averaging 25.8 min ±55.6, and had slightly more REM sleep (+2.6 ± 6.6 min).

### 2.2 Inter‐Scorers Variability Among Sleep Specialists‐Technicians?

Another limitation of PSG concerns is manual scoring, which, despite the existence of common rules, remains subject to individual interpretation by sleep doctors and technicians, even though they are trained at the same school. The agreement rate between two scorers is generally between 0.7 and 0.8 but largely depends on sleep stage. A meta‐analysis published in 2022 compared 11 studies (7 reporting information for overall sleep scoring and 4 studies separately evaluating single sleep stages) to test the inter‐rater variability of manual PSG analyses (Lee et al. 2022). This review found that a Cohen's kappa (the measure of the extent of agreement between two scorers) for manual, overall sleep scoring is 0.76, indicating substantial agreement (95% confidence interval, 0.71–0.81; *p* < 0.001). By sleep stage, the figures were 0.70, 0.24, 0.57, 0.57, and 0.69 for the W, N1, N2, N3, and R stages, respectively. This indicates a moderate inter‐rater reliabilities for stage N2 and N3 and a very low rate for stage N1. The authors suggest that the poor agreement for stage N1 can be attributed to the persistence of sparse alpha activity during wake–sleep transition that can increase the difficulty for a proper identification of sleep onset. However, it is worth noting that, if this is correct, the assessment of sleep latency is similarly compromised by variability, given that its determination relies on the precise identification of N1 sleep stage onset.

Recently in the frame of the sleep revolution project, 10 independent scorers from seven different sleep centres score manually 50 PSG recordings (Nikkonen et al. 2024). They used the 10 scorings to calculate a majority score by taking the sleep stage that was the most scored stage for each epoch. The overall agreement for sleep staging was *κ* = 0.71 and the mean agreement with the majority score was 0.86. Perfect agreement within scorer was only found for 48% of all scored epochs. The better agreement was for REM sleep (*κ* = 0.86) and the lowest for N1 sleep (*κ* = 0.41). Scorers from the same sleep centers had the highest pairwise agreements between 0.78 and 0.85.

**FIGURE 1:**
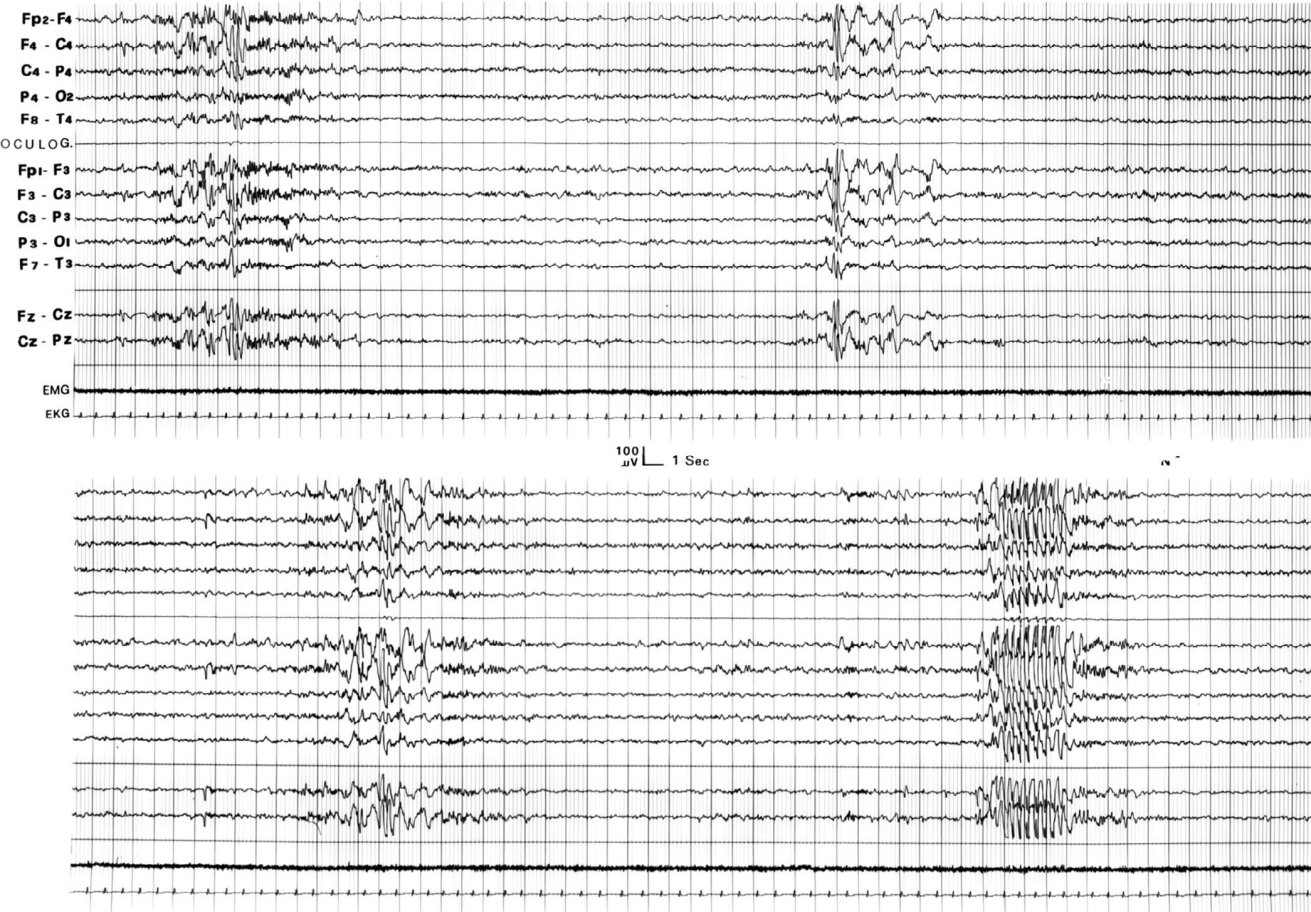
PSG and CAP report of a patient affected by diurnal and nocturnal epilepsy. At macrosructural level its sleep is unremarkable (see upper part of the figure for sleep hypnogram), but CAP (lower part of the figure) allows the identification of an impressive increase in the NREM oscillatory fluctuations, with CAP rate being strongly higher with respect to normal values (CAP rate 90.2%) indicative of unstable sleep.

This agreement rate may be even more difficult to achieve in elderly patients or those with conditions that may interfere with electroencephalography. For example, a recent study found very low agreement rates among elderly people (Chylinski et al. 2022). Twenty cognitively normal and healthy late midlife individuals' (61 ± 5 years; 10 women) night‐time sleep recordings were scored by two experts from different research laboratories and one algorithm. They computed agreements for the entire night (percentage and Cohen's *κ*) and each sleep stage. Whole‐night pairwise agreements were relatively low and ranged from 67% to 78% (*κ*, 0.54–0.67). Sensitivity across pairs of scorers proved lowest for stages N1 (8.2%–63.4%) and N3 (44.8%–99.3%). Significant differences between experts and/or algorithm were found for TST, sleep efficiency, time spent in N1/N2/N3 and wake after sleep onset (WASO) (*p* ≤ 0.005), but not for SOL, REM and slow‐wave sleep (SWS) duration (N2 + N3). In another survey focused on 15 patients with Parkinson diseases, 45 PSG nights were scored by four experienced scorers independently (West et al. 2023). Epochs with less than 75% consensus were flagged for secondary review. In secondary review of discordant epochs, two of the original scorers re‐assessed epochs, from which the final consensus stage was derived. Sleep stage agreement averaged 83.10% across all sleep stages on initial scoring, and on secondary consensus scoring 96.58%. Greatest disagreement was noted in determination of wake (33.6% of discordant epochs) and N2 (31.8% of discordant epochs). These difficulties in a proper recognition of sleep stages among the elderly become even more relevant towards patients affected by neurodegenerative disorders, such as those with alpha‐synucleinopathies, which can present a severe compromising of sleep regulatory phenomena, leading to an impressive impoverishment of NREM sleep organisation. In some cases, the derailment of NREM features is so severe that some authors suggested adopting a novel terminology, to describe what appear as ‘undifferentiated’ NREM sleep (Montini et al. 2023). See Figure 1 for a comparison between physiological N2 stage of NREM sleep and undifferentiated NREM in a patient affected by a neurodegenerative disorder.

To address the issue of low agreement among scorers regarding PSG analysis, a group of researchers in Taiwan showed how a guided learning program refreshing rules and terminology could improve the scoring in cases of mild and moderate apnea syndromes. In their experiment, 70 sleep experts from 34 sleep centers in Taiwan completed a scoring test (Montini et al. 2023) and then received 8 interactive online lectures (each lasting 30 min). Thereafter the scorers completed the second scoring test on the same data. The scorers' agreement in overall sleep stage scoring significantly increased from 74.6% to 82.3%. The proportion of scorers with an agreement of ≥ 80% increased from 20.0% (14/70) to 58.6% (41/70) after the online training program. These findings suggest that targeted educational interventions, such as guided online training, can significantly enhance scoring consistency among sleep experts, and might be considered as periodic refreshing programs (Liao et al. 2024).

### 2.3 The Loss of Stage 4

Initially, the NREM 3 stage was divided into two separate stages known as stage 3 and 4 (S3 and S4). To simplify sleep scoring system, in 2007 the American Academy of Sleep Medicine changed the classification and redefined these stages as a singular one. According to the former Rechtschaffen and Kales Sleep Staging Criteria, S3 was defined when 20%–50% of the epoch consists of high voltage (> 75 μV), low frequency (< 2 Hz) activity, while S4 when > 50% of the epoch consists of high voltage (> 75 μV) < 2 Hz delta activity. In 2007, a task force revising scoring criteria confirmed the subdivision between stage 3 and 4. The slow waves observed in these two stages are indeed associated with distinct neurophysiological phenomena (specifically, are linked to cortical oscillation for the N2 *k*‐complexes and to thalamo‐cortical and cortical networks for the delta frequencies during slow wave sleep), and also to physiological changes (e.g., tight association with the onset of growth hormone release during N3). Given the apparent absence of biological significances supporting the subdivision between S3 and S4, the task force unified the two stages in a single one, named N3 (Silber et al. 2007).

This decision has raised considerable concerns, as substantial differences exist between a stage characterised by 20% slow‐wave activity and one entirely dominated by it—differences that risk being obscured under the new unified classification. Even though sleep stages exist along a continuum rather than as discrete entities, this simplification may have constrained the thorough investigation of the features and clinical significance of deeper sleep variations, some of which bear resemblance to patterns of brain organisation observed in coma. Consequently, it may have limited important insights and considerations within the research community. This concept is particularly important especially towards sleep pathologies embedded in the slow wave sleep structure, such as NREM parasomnias.

An alternative view to describe the distinction between sleep stages, is the one proposed by Guo et al. colleagues (Guo et al. 2022), that emphasises great importance to the amount of synchronisation rather than focusing on phasic events (such as *k*‐complexes or spindles), to properly describe stages of NREM sleep as well as REM sleep. According to this synchronisation model, NREM sleep can be classified as SWS and non‐SWS, to the point that NREM sleep can be considered as an intrinsically bistable process. This distinction is not trivial as it is accompanied by concomitant fluctuations of numerous physiological activities: for instance, an increase in synchronisation is followed by stabilisation of parameters such as heart rate and respiration above a certain level, whereas deterioration of SWS is associated with a sudden increase in physiological fluctuations of these same parameters.

### 2.4 Is There Just One REM Sleep Stage?

Another debatable aspect of current sleep scoring system is the absence of a proper subdivision between phasic and tonic REM sleep stages. Although REM sleep is characterised by numerous transient neurophysiological features (e.g., REMs, muscle twitches, and changes in autonomic activity), it is usually conceptualised as a homogeneous sleep state (Simor et al. 2020). During the so‐called phasic REM periods, external information processing are attenuated, and cortical activity is detached from the surrounding environment, in contrast to tonic REM sleep in which alertness and environmental processing are partially maintained, thus resembling a wake‐like state (Simor et al. 2020; Parrino et al. 2025). More specifically, phasic REM sleep is characterised by intense cortical activity together with sensory disconnection, while tonic REM phases are quieter with respect to cortical activation but more close to wakefulness in terms of environmental alertness and arousability (Simor et al. 2020). Given the relevant biological difference between the two conditions, we question whether it might be meaningful to highlight the distinction between tonic and phasic REM in the routine reporting of this sleep stage.

In addition, in the REM sleep characterisation, the concept of periodicity that is so deeply embedded in the NREM framework is usually neglected. Whether this is due to a real absence of periodicity in this extremely unpredictable sleep stage or to our incapacity to capture its inner rhythm, is yet to define. What we know is that, at autonomic level, REM is a more chaotic stage, distinct by the domination of sympathetic nervous system activity towards parasympathetic activity, leading to more dynamic fluctuations in heart rate intervals and respiratory patterns (Mogavero et al. 2025). However, although the majority of typical periodic phenomena of NREM sleep disappear during REM sleep (e.g., CAP, PLM or Cheyne Stoke Breathing), they sometimes can sometimes persist, suggesting the necessity for further considerations (Barone 2025).

### 2.5 The Greater Proportion Rule in Sleep Scoring

Currently, if during sleep scoring we find a 30‐s epoch with coexistence of multiple sleep stages elements, AASM rules dictates that we should assign the epoch to the sleep stage that occupied the largest proportion of that 30‐s period. For instance, if a 30‐s epoch contains 15 s of N2 sleep, 10 s of N1 sleep, and 5 s of REM sleep, the epoch would be scored as N2 sleep because it represents the greatest proportion of the epoch (AASM scoring). Usually, we expect to find those ‘mixed’ epochs mostly during sleep transitions (e.g., with sleep deepening from N1 to N2 or N2 to N3, or during the sleep lightening towards REM sleep occurrence). It is intuitive to think that when sleep consolidates, the amount of these mixed sleep epochs should progressively reduce. However, in the scoring rules, there is no upper limit for these epochs and they can persist throughout the night. This can significantly obstacle the proper characterisation of sleep instability, as we loss the possibility to describe the inner complexity of these epochs. In the continued effort to simplify and standardise scoring criteria, there is a tangible risk of diminishing the diagnostic output by overlooking the complexity and variability of oscillations and intrusions that may occur within a given epoch of observation. This can be particularly relevant in some clinical scenarios, such as in narcolepsy, where the presence of altered sleep transitions is among the major findings at PSG level. To overcome this limitation, alternative methods has been proposed to properly capture the complexity of sleep, such as the hypnodensity graph, which does not enforce a single sleep stage label, but rather a membership function to each of the sleep stages, allowing more information about sleep trends to be conveyed, something that is only possible in non‐human scoring which ensure a higher (Stephansen et al. 2018).

### 2.6 The Importance of Sleep Architecture

A typical PSG report is usually full of numbers and percentages. For instance, we know that adult healthy sleepers should have around 20% of slow wave sleep and 45% of stage N2. However, these numbers do not reveal anything with respect to the reality of sleep organisation and texture. Stage N3 should predominate in the first sleep cycles, and progressively decline throughout the night, while, conversely, REM sleep is expected to increase in the second half of the night. These patterns are not merely theoretical constructs; rather, they reflect the fundamental neurophysiological organisation of sleep governed by homeostatic and circadian processes. Therefore, it is highly limitative to describe the raw percentage of sleep stages with no identification of the sleep texture in terms of hypnogram. Patients sharing the same amount of stage N3, will not have the same clinical scenario if the first present a regular subdivision of this sleep stage in the first half of the night and the second one present scattered distribution of short periods of stage N3 randomly distributed during sleep. The latter might have something disturbing the consolidation of this sleep stage warranting further consideration.

Even stage N2, which typically dominates throughout the night, should not be considered a uniform stage, as it can be distinguished into a ‘quiet’ N2 period, when it preceded SWS onset, and a more ‘active’ sleep N2 stage, when it preceded REM sleep onset. This difference has been confirmed by autonomic and endocrinological evaluations showing that both the adrenocorticotropic system and the renin‐angiotensin system, together with the cardiovascular system faced different changes during stage N2, depending on the following sleep stage (Brandenberger et al. 2005).

Given that, PSG is a diagnostic tool; its interpretation must go beyond quantitative summaries. A critical and holistic analysis of sleep architecture—such as evaluating the hypnogram and sleep stage continuity—is essential to uncover patterns that raw numbers alone cannot reveal.

### 2.7 PSG or Video‐PSG?

The full version of PSG is video‐PSG, which should preferably be performed in a hospital setting with high‐resolution cameras. The use of video support is warranted by the frequent presence of motor phenomena in a wide range of sleep disorders, which are often difficult to distinguish based solely on the patient's or caregivers' clinical history. A nocturnal hypermotor episode may correspond to an event of sleepwalking, a REM parasomnia, an epileptic seizure, or, in some cases, an arousal associated with apnea or periodic limb movement. Scalp EEG recordings and standard cardiorespiratory sensors may not provide sufficient diagnostic clarity on their own. Video recording thus plays a crucial role in supplementing the semiological characterisation of events, enabling a more accurate differential diagnosis. Video‐PSG analysis is currently considered among the most reliable tool in the differential diagnosis between NREM parasomnias and SHE, due to distinct motor manifestations of the two conditions (Loddo et al. 2020, 2018). The relevance of video assessment in a clinical setting should not be surprising, even in the current era of digitalization and advanced technology. Semiology remains one of the foundational pillars of modern medicine, and sleep medicine is no exception.

Typically, video in v‐PSG is used essentially to visualise patients during sleep, however, in the latest years, thank to impressive growth in engineering research, video have been proposed to assess signals derived from pulse, breath and motions, to obtain a contactless automated sleep stages scoring system, with moderate accuracy (ranging from 68 to up to 80%) (Wang et al. 2025; van Meulen et al. 2023). The possibility to gain a contactless analysis for sleep has been investigated as PSG is, by nature, an intrusive exam and, as already explained in a previous subchapter in this paper, this can lower its diagnostic reliability (FNE, patients' discomfort).

Thus, integrating PSG recordings with video monitoring should be considered a core component of the diagnostic process, rather than a secondary aid, due to the substantial amount of information it can yield, whether through direct visual assessment or through more sophisticated, engineering‐driven analytical methods.

## 3 Costs and Burden of Polysomnography in the Lab and at Home. Transition to Wearables

### 3.1 Costs and Burden of PSG

In addition to these technical limitations, it is clear that performing PSG in a sleep laboratory, or even at home, represents a significant economic burden for both clinicians and patients (Pendharkar et al. 2024; Torres‐Granados et al. 2023). Countries' social security authorities to perform these procedures in accordance with international consensus recommendations accredit sleep laboratories. Medical and technical staff are trained in the performance of PSG. The equipment is relatively expensive. The time required to attach the electrodes, inform patients, and read the tracings from a night of PSG is estimated at 2–3 h of specialised staff time, and can be even more in complicated cases (e.g., elderly, neurodegenerative disorders). As already outlined, recently, in the US as in many countries, PSG can be performed on an outpatient basis (Furlan et al. 2025). However, in some cases, particularly when investigating NREM or REM parasomnias or sleep epilepsies, but also for rare hypersomnia disorders, laboratory recording with video surveillance requires hospitalisation for one night (Frandsen et al. 2021). Sleep disorders are therefore associated with significantly higher rates of health care utilisation and expenditures than people with no sleep disorders. Using a conservative prevalence estimate of sleep disorders in the United States, Huyett & Bhattacharyya calculated an overall incremental health care costs of sleep disorders of approximately $94.9 billion in 2017 (Huyett and Bhattacharyya 2021).

### 3.2 The Place of Wearables

In this context, careful consideration must be given to the development of a wide range of connected devices designed to measure sleep. The first of these was actigraphy, which is now recognised as a valid tool in the diagnosis of circadian sleep disorders: delayed or advanced sleep phase, shift work disorders, Non‐24, jet lag (Gillett et al. 2021). However, actigraphy is also well validated in insomnia and in narcolepsy, particularly because it allows the reality of patients' sleep–wake rhythms to be assessed over a period of several days or even weeks, making it is easier to recognised misperception or insufficient sleep syndrome (Natale et al. 2014; Banfi et al. 2021; Leger et al. 2018).

Furthermore, most connected sleep devices today are not medical devices, but rather bracelets, headbands, textile sensors, or rings aimed at people who are conscious of their well‐being or performance. These devices provide increasingly accurate information about time in bed (TIB), TST, WASO, sleep schedules, napping, heart rate, respiratory rate, blood oxygen saturation, breathing disturbances and, sometimes, even sleep stages.

These non‐intrusive devices have the potential to facilitate long‐term sleep monitoring, are broadly accessible, and may contribute to the redefinition of normative sleep parameters, which are likely subject to change in response to evolving environmental and behavioural factors such as increased artificial light exposure, dietary shifts, technological advancement, and modern work patterns.

Several systematic reviews have recently compared the most common of these connected devices with PSG (Lee et al. 2023; Miller et al. 2022; Svensson et al. 2024; Schyvens et al. 2024; Willoughby et al. 2024). It is not the purpose of this article to discuss these results in detail, but these reviews confirm the growing quality of these devices, whose miniaturisation and use of AI algorithms increase their relevance. In light of these developments, it is advisable to refer to the World Sleep Society's recommendations for the use of these connected devices (Chee et al. 2025). These recommendations are intended for manufacturers and clinicians. They differentiate between their use in healthy adults and those with sleep disorders. They also address the use of digital sleep data and medico‐legal aspects in a relevant manner. In summary, it is likely that in the future, these connected devices will play a role in the screening and maybe even in the diagnostic path of patients with suspected sleep disorders.

## 4 PSG in the Future: Clinical and Research Perspectives

Despite its technical limitations and the cost associated with its implementation, we remain convinced that PSG is very valuable in diagnosing major sleep disorders: insomnia, sleep‐related breathing disorders, hypersomnia, epilepsy and parasomnia. It provides information about the disorder that are sometimes overlooked by clinicians and can greatly benefit from artificial intelligence (AI) techniques to provide patients with more comprehensive information.

### 4.1 PSG in the Future of Insomnia

The definition of insomnia is currently based on subjective complaints, and PSG is not mandatory to confirm the diagnosis, unless there is doubt about the presence of coexistent sleep pathologies (e.g., apnea or periodic leg movements). However, for several years now, with the greater ease of testing, many clinicians have recommended PSG in difficult cases and when treatment is ineffective. This practice is included in expert consensus guidelines (Riemann et al. 2023).

### 4.2 Revisiting PSG of Insomnia and Sleep Misperception

Recently, several studies have looked at the computational analysis of PSG in people complaining of insomnia with or without sleep misperception (SSM). They showed how an in‐depth analysis of PSG features, moving beyond the mere sleep stages classification, can provide way more info than the simple macroscopic results we often stick to.

A PSG study comparing chronic insomnia patients (with and without misperception) with healthy sleepers documented that, adopting AI, is it possible to differentiate good sleepers from real insomniac patients. In details, PSG of 347 patients with chronic insomnia, including 59 with sleep state misperception (SSM) and 288 without (INS) were compared to the ones of 89 controls—the good sleepers (GS) (Andrillon et al. 2020). The authors retained three series of features: (1) macroscopic indexes derived from the conventional hypnogram, (2) mesoscopic indexes extracted from the EEG spectrum, (3) sleep microstructure (slow waves, spindles). Therefore, adopting a supervised algorithms, the authors aimed to differentiate patients from GS.

The macroscopic level confirmed that insomniac patients without SSM spent less time asleep than good sleepers (TST: 361 min ±5.1 vs. 406 min ±5.4; *p* = 4.0.10–6) and had a decreased sleep efficiency (72.0% ± 0.8% vs. 79.7% ± 1.1%; *p* = 2.1.10–6). INS group spent also a larger proportion of time awake during the night (27.8% ± 0.8% vs. 20.3% ± 1.1%; *p* = 4.1.10–6) and less time in NREM2 (41.7% ± 0.7% vs. 47.1% ± 0.9%; *p* = 3.8.10–5) as well as REM sleep (12.9% ± 0.3% vs. 15.0% ± 0.5%; *p* = 0.001). These results confirm the rather archetypal profile of the patients from the INS group.

Interestingly, the same features failed to evidence any sleep disturbance in the insomnia group with SSM. These patients appeared to actually sleep better than controls, with increased sleep efficiency (88.4% ± 0.8% vs. 79.7% ± 1.1%; *p* = 2.4.10–8), decreased proportion of wakefulness during the night (11.7% ± 0.8% vs. 20.3% ± 1.1%; *p* = 2.2.10–8) and increased proportion of NREM3 stage (19.5% ± 1.2% vs. 15.1% ± 0.7%; *p* = 4.4.10–4). Yet, based on subjective complaints, these patients were diagnosed with insomnia and had with standard PSG criteria a better sleep than healthy sleepers.

Another interesting difference emerge with respect to sleep transitions. The mispercetionists patients curiously presented a higher tendency for sleep transitions from wakefulness to stage N2 compared to objectively tested insomnia patients and healthy sleepers, confirming the SSM paradox, whereby subjective complaints contrast with a better‐than‐usual transition to sleep.

Although at conventional level these paradoxical differences emerge, when evaluating sleep with spectrum analysis methods then the spectrum of the insomnia subtypes (INS and SSM) significantly overlap, although INS patients showed an increased level of alpha/theta oscillations compared to SSM (post hoc cluster: [0, 30]%, *p* cluster < 0.005). These results alone could positively help overcome the discrepancy between objective and subjective assessments of insomnia: SSM patients no longer look like good sleepers but are much more similar to INS patients when examining the spectral features of the EEG signal.

Furthermore, important difference between good sleepers and insomnia patients was observed with respect to spindle representation. In details, spindle frequency varied across groups in stage N2, with faster spindles in both INS and SSM patients. Importantly, there were significant differences between insomnia subtypes in terms of spindle density and frequency. SSM patients showed larger number of sleep spindles compared to INS patients, in stage N2 and N3. This observed increase in the spindle density and frequency could be a direct consequence of the reduction of the representation and stability of SWS, lastly reflecting an increase in cortical excitability during sleep.

### 4.3 Sleep Entropy

Going one‐step further in the dynamic analysis of PSGs of patients with INS and SSM, a novel parameter to detect hypnodensities (HD) within sleep was also suggested (Herzog et al. 2025). HD is a new method based on neural network analysis of sleep that does not indicate a single sleep stage label for each epoch, but rather defines a membership function to each of the sleep stages, aiming to inform more specifically on sleep trends. Entropy is a function of the full HD that measures the proximity to a uniform distribution, thus providing more information than just measuring the highest probability on the HD. As high HD entropy indicates a high level of intrusion (proximity to a uniform distribution), high entropy during wakefulness implies sleep intrusions, while high entropy during sleep implies wakefulness or intrusions from other sleep stages. Consistent with the previous results, we found a significant increase of entropy during wakefulness in both insomnia groups (with and without SOSD) compared to good sleepers, and in SOSD+ compared to SOSD− (Herzog et al. 2025). This method has been already applied to narcolepsy type I and insomnia, with interesting results (Stephansen et al. 2018; Brandenberger et al. 2005; Loddo et al. 2020, 2018; Wang et al. 2025; van Meulen et al. 2023; Pendharkar et al. 2024; Torres‐Granados et al. 2023; Furlan et al. 2025; Frandsen et al. 2021; Huyett and Bhattacharyya 2021; Gillett et al. 2021; Natale et al. 2014; Banfi et al. 2021; Leger et al. 2018; Lee et al. 2023; Miller et al. 2022; Svensson et al. 2024; Schyvens et al. 2024; Willoughby et al. 2024; Chee et al. 2025; Riemann et al. 2023; Andrillon et al. 2020; Herzog et al. 2025). For instance, adopting hypnodensity, Herzog et al., analysed sleep intrusions and instability in PSG recordings from a large clinical database including 624 patients with insomnia without subjective‐objective sleep discrepancy (SOSD), 199 patients with insomnia and SOSD, and 104 controls healthy controls. In this study, sleep and wake intrusions were defined respectively as the probability of wakefulness during sleep epochs and the probability of sleep during wake epochs. In this analysis both SOSD− and SOSD+ insomnia sub‐types had sleep intrusions during wakefulness, but in SOSD+ we found no wake intrusions during sleep (Herzog et al. 2025).

Thus, this approach on HD does not only lead to good classification performance but also allows us to interpret the underlying features, shedding a new light on the physiopathology of insomnia.

### 4.4 Insomnia, PSG and Depression

Sleep abnormalities are core symptoms in numerous mood disorders, including major depression and bipolar disorder, Insomnia for instance is a characteristic manifestation of depression and, although PSG is still relatively uncommon in psychiatric practice, REM pressure, assessed by REM latency and the percentage of REM sleep, is a considered a reliable electrophysiological biomarker of depression. Adopting computerised spectral analysis recent studies investigated the presence of REM‐related biomarkers of depression at PSG level. (Solelhac et al. 2024) studied the PSG characteristics of 717 subjects from the Hypnolaus cohort in the general Swiss population with incident major depressive episodes (MDD) in a general community‐dwelling cohort. Beside the conventional PSG features like TST, WASO, SOL, SE, REM latency and the percentages of REM and non REM sleep, the authors extracted two novel features: (1) REM density, corresponding to the number of REMs (automatically detected) per minute of REM sleep; (2) absolute delta power spectral in the first 50% of the night. According to this population‐based study, elevated REM density values are significantly associated with MDD in men (HR 1.270, 95% CI 1.064–1.516) and higher delta power values are associated with a decreased incidence of MDD in women (HR 0.674, 95% CI 0.463–0.981). The latter result confirmed previous investigation demonstrating that delta power was reduced on the first half of the night in depression (Svetnik et al. 2017). Notably, the authors underlined that sex differences cannot be utterly explained by their results as the median age of enrolled subjects was over 50 years (menopausal status for most of women), limiting the generalizability of their findings.

A recently published systematic review with meta‐analysis analysing sleep abnormalities in bipolar disorder attested that during the depressive phase patients show higher REM percentages, while manic/mixed phases exhibit shorter TST, lower SE, and longer SOL (Marchetti et al. 2025).

In this framework, PSG features, especially when evaluated with the support of computerised based analysis could be new tools in the future of PSG which can serve as essential tools for bridging the gap between the field of sleep medicine and that of psychiatry. It could help in narrowing the boundaries between these areas, which are often closely linked at a neurobiological level, but not always easy to characterise clinically.

## 5 An Alternative Dynamic Analysis of PSG: Cyclic Alternating Pattern (CAP) and Odd Ratio Products (ORP)

With a visionary approach combining physiology and dynamic perception of sleep states, a research team in the University Hospital of Parma, Italy, had proposed since 1985 an alternative, more dynamic and holistic analysis of sleep, by observing the multisistemic periodicity present at PSG level in the form of cyclic alternative pattern (CAP) (Terzano et al. 1985).

The CAP is characterised by the periodic recurrence of 2–60 s alternate electroencephalogram (EEG) patterns occurring during NREM sleep. It is a pattern of spontaneous cortical activity that occurs even in the absence of sensory stimulation, although can be modified from external stimuli (Terzano et al. 1988) (e.g., noise). CAP mirrors the reorganisation of the sleeping brain challenged by the modification of environmental conditions and it is characterised by periodic abnormal electrocortical activity that recurs with a frequency of up to one minute (Chee et al. 2025). It is considered the EEG marker of unstable sleep (Parrino et al. 2012). CAP does not occur during REM sleep. CAP is organised into sequences of successive cycles composed of two phases, A and B. Phase A involves lumps of phasic events, while the following phase B reflect the return to background activity. Phase A subtypes of CAP (named A1, A2 and A3) allow adaptive adjustments of ongoing brain activity to internal and external inputs (Terzano et al. 1985; Parrino et al. 2012). For instance, subtypes A1 typically exert a sleep‐promoting function and dominate in the first half of the night and during the descending slope of sleep cycles, while A2 and A3 usually contribute to lighten sleep towards arousals or REM sleep, are located especially in the ascending slope of sleep cycles and prevail in the second part of the night. The CAP framework reflects but it not limited to EEG activity, as its oscillatory pattern is tightly linked to cardiovascular, respiratory and autonomic functions, including potentially cerebral spinal fluid fluctuations (Mutti et al. 2024). The latter association received indirect confirmations by functional MRI investigations revealing that BOLD signal in the cortex oscillates at 0.05 Hz (1 cycle every 20 s) during the first stages of drowsiness, the same time domain of CAP (Gaez et al. 2025). Interaction between CAP, neurovegetative fluctuations and motor events determine the pathophysiology of several sleep disorders like OSA, one of the main representatives of CAP phase B‐disorders.

CAP is also the “master clock” that accompanies the stage transitions: a significant increase in the CAP subtypes A2 and A3 is expected before the REM sleep onset, while CAP oscillations are usually reduced in the minutes following REM sleep.

CAP can also be use to categorised sleep and neurological disorders: it is typically decreased in narcolepsy, chronic utilisation of hypnotic drugs, Parkinson disease, multiple system atrophy, amyotrophic lateral sclerosis. It can be adopted to measure efficacy in OSA under CPAP treatment, and typically declines during night‐time recovery sleep after prolonged sleep deprivation (Andrillon et al. 2020). Conversely, CAP is impressively high in patients affected by NREM sleep parasomnia, nocturnal epilepsy, moderate‐to‐severe OSA and periodic limb movements disorders (Mutti et al. 2023; Parrino et al. 2022). In certain cases, the CAP can capture the true fragility and instability of NREM sleep, aspects that conventional PSG may fail to adequately reveal.

There is a relationship present between CAP and arousals that allows for adjustments of vigilance during sleep. Although according to AASM scoring criteria, fast arousals are considered as detrimental with respect to sleep continuity, numerous investigations attested that are natural guests of human sleep serving as channels of communication with the outside world. From an evolutionary standpoint, it would not be adaptive for sleep to entail a complete disconnection from the external environment, as this would excessively increase the risk of exposure to threats. Accordingly, both rapid and slow arousals, integrated within the electrophysiological construct of the CAP, serve to maintain a smoother continuity between sleep and wakefulness. These arousals modulate the transitions between deeper and lighter sleep stages throughout the night and act as signals of potential intrusions, whether originating from external stimuli or internal processes, thereby functioning as true sentinels of sleep (Parrino and Vaudano 2018).

If there is a failure in this relationship between arousals and sleep, sleep disorders may develop.

More recently, CAP have been integrated into computerised sleep analysis programs, allowing a more dynamic approach to sleep that takes into account external influences and internal automatic rhythms (Herzog et al. 2025). A recent systematic review on 35 articles on CAP had concluded indeed that studying CAP is highly arduous for human experts; thus, developing automated systems for assessing CAP is gaining momentum (Mendonça et al. 2023). Developing new techniques for automated CAP detection installed in clinical setups is essential. This paper presents the algorithms and methods in the literature for the automatic assessment of CAP and the development of CAP‐based sleep markers that may enhance sleep quality assessment, helping diagnose sleep disorders. It could be also crucial to better understand sleep disturbances associated with autonomic changes with the help of CAPs in chronic diseases like Parkinson Diseases or systemic sclerosis (Cheng et al. 2025; Gök et al. 2025).

The further development of automated CAP analysis of PSGs should allow for a more accurate diagnosis of sleep disorders in the future.

## 6 ORP: Odds Ratio Product

ORP is also a visionary and dynamic method of visualising sleep that was proposed over 10 years ago by Younes (Younes et al. 2021), but whose digitization and machine learning methods of analysis have enabled an easier and more widespread use in recent years.

**FIGURE 2:**
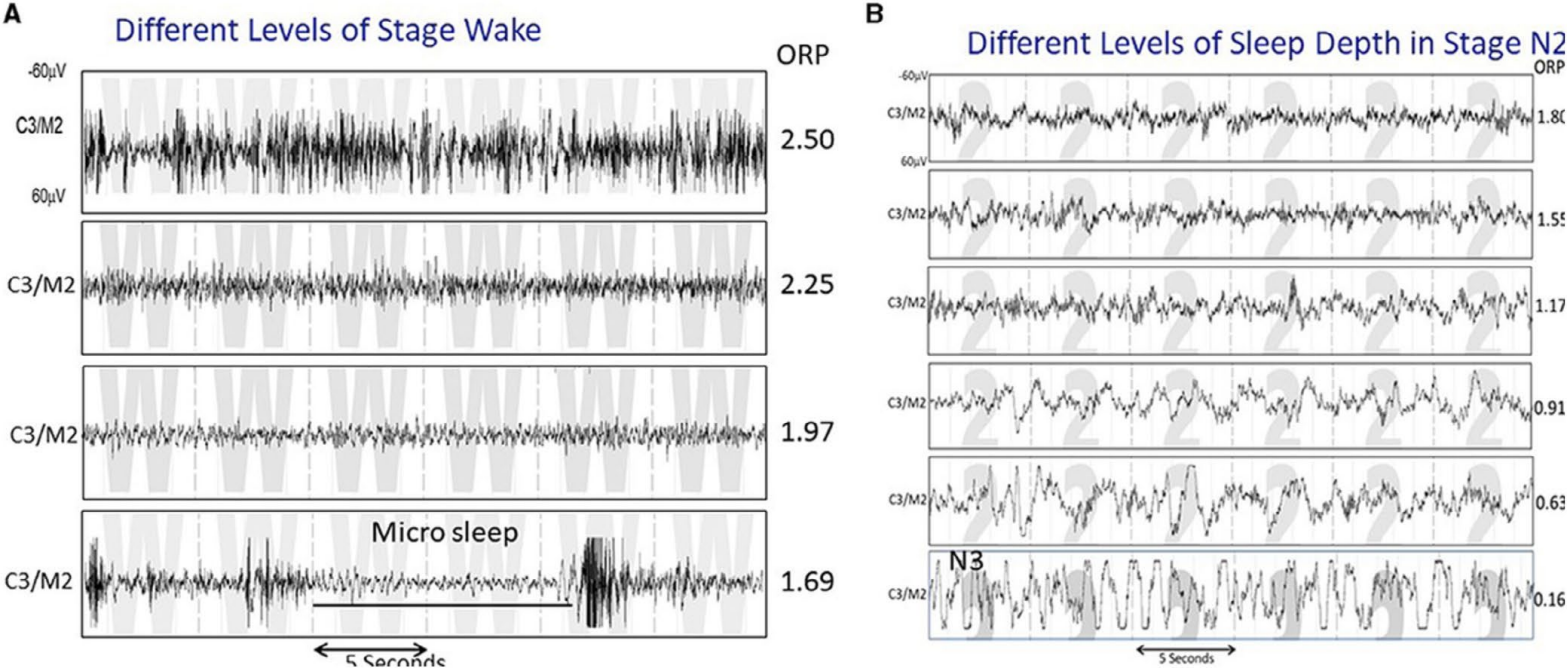
ORP: (A) Four 30‐s strips of EEG tracings all staged as wake, illustrating various states between full wakefulness (top tracing) and near sleep. The ORP values reflect these differences. (B) Five 30‐s strips staged as N2 but showing a variety of patterns that range from one that reflects very light sleep (top panel) to one that is very similar to stage N3 except that the total duration of delta waves is < 20% of the epoch (From reference: Younes M, Azarbarzin A, Reid M, Mazzotti DR, Redline S.). Characteristics and reproducibility of novel sleep EEG biomarkers and their variation with sleep apnea and insomnia in a large community‐based cohort. Sleep. (67).

By definition: the odds ratio (ORP) is a continuous measure of sleep depth ranging from 0 (very deep sleep) to 2.5 (fully awake). The major advantage of this method is that ORP consents to disclose different levels of stage wake (sleep propensity) and different sleep depths within the same sleep stage (Younes 2023). A fast Fourier transform is applied to all EEG values in non‐overlapping 3‐s periods. The total power in each of the 4 frequency ranges, between 0.33 and 35.0 Hz, is calculated. The power in each frequency range is given a score between 0 and 9 based on its position in the power range (in the corresponding frequency) observed in 56 clinical polysomnograms (PSGs). ORP can be used to identify differences in sleep depth between subjects. It is also proposed to evaluate the same subjects in different circumstances, when these differences are not detectable by the conventional method. It also identifies different depths of sleep within REM sleep, which may have implications for disorders occurring during this phase. ORP per epoch can be graphed over the entire night or as average values in conventional sleep stages. In addition, ORP can be expressed as a percentage of recording time in specific ORP ranges (e.g., deciles of the total ORP range). This representation offers unique research opportunities to identify various dynamic mechanisms and potential treatments for different sleep disorders (Figure 2).

Several recent studies have validated ORP in large groups and highlighted its relevance and ability to differentiate phenotypes in insomnia, for example. Thus, a study comparing PSGs from 333 controls and 194 subjects with insomnia explores EEG markers of insomnia by integrating subjective reports and objective physiological markers, particularly ORP and spectral characteristics. It aims to describe the phenotypic profiles of patients based on subjective complaints and objective findings (Lee et al. 2025). The research’ goal is to determine whether groups defined by weekly frequency and daily duration of symptoms show different distribution patterns and which physiological characteristics best distinguish insomnia patients from controls.

The results suggest that ORP, as a dependent variable, captures the most significant similarities between independent variables in the overall model. For example, high beta power in insomnia patients indicates increased cortical arousal, supporting the hypothesis that insomnia is a hyperarousal disorder. Another study used ORP to describe 15,940 PSG of 13,004 patients (average age of 54 years old) from one hospital site. Patients were tested cognitively and their sleep was analysed using respiratory parameters and leg movements (Meulenbrugge et al. 2025). Correlations with sleep stages, arousal Index and clinical variables were calculated. ORP showed a strong linear correlation with arousal probability (Pearson's *r* = *r* = 0.923). ORP also reflected expected decreases in sleep depth with advancing age and demonstrated that females have significantly deeper sleep than males across several stages. The authors concluded that ORP provide a nuanced understanding of sleep architecture with physiological and pathological implications. Lastly (Hanif et al. 2025), retrospectively analysed 9750 patients in four groups: (1) 1152 controls; (2) 2395 with chronic insomnia; (3) 2297 with OSA; and (4) 3906 with COMISA. ORP, computed every 3 s from polysomnography, was analysed alongside sleep metrics, comorbidities, and sleep habits. Machine learning models classified COMISA, OSA, and CIN as distinct clinical groups. They concluded that ORP‐derived features showed stronger associations with COMISA than traditional sleep metrics (except N3 latency). Independent objective predictors of COMISA included male sex (OR = 1.31, 95% CI = [1.16, 1.47]), BMI (1.27, [1.25, 1.29]), N3 latency (1.21, [1.13, 1.29]), age (1.17, [1.16, 1.19]), peak ORP during spontaneous arousals (1.12, [1.01, 1.25]), and time in ORP decile 7 (1.10, [1.07, 1.13]). Subjective predictors included depression, hypertension, allergy, headache, sleep aid/alcohol use, sleepiness, and lower sleep duration. Machine learning achieved overall accuracy of 61.2% (*p* < 0.05), with sensitivity of 71% for COMISA and less accurately 65% for OSA and 43% for chronic insomnia. ORP is therefore an innovative complementary analysis for assessing sleep depth and allows for dynamic sleep classification, including according to gender and age.

## 7 Is There a Role for PSG in the Local Sleep Analysis?

Another important consideration is regarding the possibility to explore sleep at local level with non‐invasive techniques such as PSG. It is well known that sleep exists as global and local phenomenon, with important biological consequences. In the latest years growing evidences supported the concept of both sleep and wake regulated locally, and that sleep‐ and wake‐like activity can often co‐occur across distinct brain areas (Bernardi et al. 2020). However, the local nature of sleep nowadays can be confirmed essentially via invasive techniques, such as stereo‐EEG or advanced technology such as high‐density EEG, that have limited applicability in everyday clinical practice. A study analysing sleep states with depth electrodes revealed an important issue: neocortex and hippocampus could be (and frequently are) in nonsimultaneous states during sleep. On average, one‐third of the night these two brain areas are divergent in terms of sleep/wake stage and the hippocampus often led in asynchronous state transitions (Jang et al. 2022). If the concept of uni‐hemispheric or local sleep as been widely accepted for numerous animal species (seals, dolphins, rodents and others), the evidence supporting them in human research is somehow less consistent. This can be attribute the difficulty in capturing the complexity of brain states during sleep measuring only surface cortical oscillations with PSG.

Give the importance that the phenomenon of local sleep during wakefulness can have at behavioural level and for general health, we believe that in the future, great efforts should be made to achieve reliable non‐invasive methods to explore it in healthy sleepers and sleep disordered patients.

## 8 Conclusion Perspectives

From dynamic analysis to artificial intelligence, automatic polysomnography analysis is at the heart of the debate. We recently compared the performance of five automatic sleep analysis algorithms (Mutti et al. 2023). Their results are already very impressive in detecting sleep stages and microstructure elements. We discussed that in the future, it will be up to clinicians to decide which type of algorithm to use based on the sleep disorder, sociodemographic variables, and comorbidities. Each polysomnography can then be detailed and described in terms of its macrostructure and microstructure, and the information given to patients will be much more relevant than it is today. CAP and ORP will be able to add the dynamic dimension.

Furthermore, this area of research is very active, and we know that in the near future many other “open source” algorithms will be developed specifically for each sleep disorder.

This is a new approach that will certainly also be appropriate for sleep technicians, who will need tomorrow to master these new data learning techniques and contribute to the dimensional analysis of these PSGs.

## Author Contributions

**Damien Leger:** conceptualization, methodology, investigation, validation, visualization, writing – review and editing, writing – original draft, supervision. **Carlotta Mutti:** conceptualization, investigation, writing – original draft, writing – review and editing, validation. **Alexandre Rouen:** investigation, writing – original draft, writing – review and editing, methodology. **Liborio Parrino:** conceptualization, investigation, writing – original draft, validation, writing – review and editing, supervision.

## Conflicts of Interest

Dr Leger reports support to himself or his institution from Actelio, Bioserenity, Sanofi, Vanda, Linde, SOS oxygene including board membership, consulting or advisory roles, and travel reimbursment. The other authors declare no conflicts of interest.

## Data Availability

Research data are not shared.
